# Supplementary material for: Identifying meta-research with researchers as study subjects: Protocol for a scoping review
Source: PLoS One. 2024 May 20;19(5):e0303905. doi: 10.1371/journal.pone.0303905 (PMC11104640; doi:10.1371/journal.pone.0303905)
Supplement: S2 Table — (DOCX) [file pone.0303905.s002.docx]

**Identifying meta-research with researchers as study subjects: protocol for a scoping review**

**Table S2. Testing of Search Strategy Sensitivity on Initial Set of Eligible Studies**

| **Study Article** | **Article Detected?** |
| --- | --- |
| Brownson RC, Jacobs JA, Tabak RG, Hoehner CM, Stamatakis KA. Designing for dissemination among public health researchers: findings from a national survey in the United States. Am J Public Health. 2013;103(9):1693-1699. doi:10.2105/AJPH.2012.301165 | Yes |
| Stevens ER, Shelley D, Boden-Albala B. Unrecognized implementation science engagement among health researchers in the USA: a national survey [published correction appears in Implement Sci Commun. 2020 Jul 15;1:65]. Implement Sci Commun. 2020;1:39. Published 2020 Mar 30. doi:10.1186/s43058-020-00027-3 | Yes |
| Stevens ER, Shelley D, Boden-Albala B. Perceptions of barriers and facilitators to engaging in implementation science: a qualitative study. Public Health. 2020;185:318-323. doi:10.1016/j.puhe.2020.06.016 | Yes |
| Grande D, Gollust SE, Pany M, et al. Translating research for health policy: researchers' perceptions and use of social media. Health Aff (Millwood). 2014;33(7):1278-1285. doi:10.1377/hlthaff.2014.0300 | Yes |
| Narendorf SC, Small E, Cardoso JA, Wagner RW, Jennings SW. Managing and Mentoring: Experiences of Assistant Professors in Working with Research Assistants. Soc Work Res. 2016;40(1):19-30. doi:10.1093/swr/svv037 | Yes |
| Benjamin DM, Hey SP, MacPherson A, et al. Principal investigators over-optimistically forecast scientific and operational outcomes for clinical trials. PLoS One. 2022;17(2):e0262862. Published 2022 Feb 8. doi:10.1371/journal.pone.0262862 | Yes |
| Miller FA, Mentzakis E, Axler R, et al. Do canadian researchers and the lay public prioritize biomedical research outcomes equally? A choice experiment. Acad Med. 2013;88(4):519-526. doi:10.1097/ACM.0b013e31828577fe | Yes |
| Holliday E, Griffith KA, De Castro R, Stewart A, Ubel P, Jagsi R. Gender differences in resources and negotiation among highly motivated physician-scientists. J Gen Intern Med. 2015;30(4):401-407. doi:10.1007/s11606-014-2988-5 | Yes |
| Huybers T, Greene B, Rohr DH. Academic research integrity: Exploring researchers' perceptions of responsibilities and enablers. Account Res. 2020;27(3):146-177. doi:10.1080/08989621.2020.1732824 | Yes |
| Shamsi A, Lund B, Mansourzadeh MJ. Gender Disparities Among Highly Cited Researchers in Biomedicine, 2014-2020. JAMA Netw Open. 2022;5(1):e2142513. Published 2022 Jan 4. doi:10.1001/jamanetworkopen.2021.42513 | Yes |
| Deardorff A. Why do biomedical researchers learn to program? An exploratory investigation. J Med Libr Assoc. 2020;108(1):29-35. doi:10.5195/jmla.2020.819 | Yes |
| Gu C, Ye M, Wang X, Yang M, Wang H, Khoshnood K. Nurse researchers' perspectives on research ethics in China. Nurs Ethics. 2019;26(3):798-808. doi:10.1177/0969733017720848 | Yes |
| Stvilia B, Wu S, Lee DJ. Researchers' participation in and motivations for engaging with research information management systems. PLoS One. 2018;13(2):e0193459. Published 2018 Feb 23. doi:10.1371/journal.pone.0193459 | Yes |
| Stevens ER, Shelley D, Boden-Albala B. Barriers to engagement in implementation science research: a national survey. Transl Behav Med. 2021;11(2):408-418. doi:10.1093/tbm/ibz193 | No |
| Schofield DJ, Meachem S, West C, Kavallaris M, Callander EJ. A crisis in the making? Education, ageing populations and the future of the medical research workforce. Med Educ. 2011;45(2):200-207. doi:10.1111/j.1365-2923.2010.03785.x | No |
| Xaverius PK, Homan S, Nickelson PF, Tenkku LE. Disparities rank high in prioritized research, systems and service delivery needs in Missouri. Matern Child Health J. 2007;11(5):511-516. doi:10.1007/s10995-007-0187-2 | No |
| Jolliffe L, Hoffmann T, Laver K, McCluskey A, Lannin NA. Stroke rehabilitation research translation in Australia: a survey of clinical trialists. Disabil Rehabil. 2022;44(10):2131-2137. doi:10.1080/09638288.2020.1807619 | No |
| Sullivan A, Murray EJ, Corlin L. Academic training of authors publishing in high-impact epidemiology and clinical journals. PLoS One. 2022;17(7):e0271159. Published 2022 Jul 29. doi:10.1371/journal.pone.0271159 | No |
| McGee PL. A descriptive study of faculty-to-faculty incivility in nursing programs in the United States. J Prof Nurs. 2021;37(1):93-100. doi:10.1016/j.profnurs.2020.07.004 | No |
| Nicholson J, McCrillis A, Williams JD. Collaboration challenges in systematic reviews: a survey of health sciences librarians. J Med Libr Assoc. 2017;105(4):385-393. doi:10.5195/jmla.2017.176 | No |
| **Yield** | **13/20 = 65%** |
