# Supplementary material for: Identifying meta-research with researchers as study subjects: Protocol for a scoping review
Source: PLoS One. 2024 May 20;19(5):e0303905. doi: 10.1371/journal.pone.0303905 (PMC11104640; doi:10.1371/journal.pone.0303905)
Supplement: S3 Table — (DOCX) [file pone.0303905.s003.docx]

**Identifying meta-research with researchers as study subjects: protocol for a scoping review**

**Table S3. Core Search Strategy (PubMed):**

(("clinician-scientist engaged" [title/abstract:~2] OR "clinician-scientist engagement" [title/abstract:~2] OR "clinician-scientist engaging" [title/abstract:~2] OR "clinician-scientist experiences" [title/abstract:~2] OR "clinician-scientist involved" [title/abstract:~2] OR "clinician-scientist involvement" [title/abstract:~2] OR "clinician-scientist participate" [title/abstract:~2] OR "clinician-scientist participated" [title/abstract:~2] OR "clinician-scientist participating" [title/abstract:~2] OR "clinician-scientist participation" [title/abstract:~2] OR "clinician-scientist perceptions" [title/abstract:~2] OR "clinician-scientist responded" [title/abstract:~2] OR "clinician-scientist responding" [title/abstract:~2] OR "clinician-scientist responses" [title/abstract: ~2] OR "clinician-scientist's experiences" [title/abstract:~2] OR "clinician-scientist's perceptions" [title/abstract:~2] OR "clinician-scientist's responses" [title/abstract: ~2] OR "clinician-scientists engaged" [title/abstract:~2] OR "clinician-scientists engagement" [title/abstract:~2] OR "clinician-scientists engaging" [title/abstract:~2] OR "clinician-scientists experiences" [title/abstract:~2] OR "clinician-scientists involved" [title/abstract:~2] OR "clinician-scientists involvement" [title/abstract:~2] OR "clinician-scientists participated" [title/abstract:~2] OR "clinician-scientists participating" [title/abstract:~2] OR "clinician-scientists participation" [title/abstract:~2] OR "clinician-scientists perceptions" [title/abstract:~2] OR "clinician-scientists responded" [title/abstract:~2] OR "clinician-scientists responding" [title/abstract:~2] OR "clinician-scientists responses" [title/abstract: ~2] OR "investigator engaged" [title/abstract:~2] OR "investigator engagement" [title/abstract:~2] OR "investigator engaging" [title/abstract:~2] OR "investigator experiences" [title/abstract:~2] OR "investigator involved" [title/abstract:~2] OR "investigator involvement" [title/abstract:~2] OR "investigator participated" [title/abstract:~2] OR "investigator participating" [title/abstract:~2] OR "investigator participation" [title/abstract:~2] OR "investigator perceptions" [title/abstract:~2] OR "investigator responded" [title/abstract:~2] OR "investigator responding" [title/abstract:~2] OR "investigator responses" [title/abstract: ~2] OR "investigator's experiences" [title/abstract:~2] OR "investigator's perceptions" [title/abstract:~2] OR "investigator's responses" [title/abstract: ~2] OR "investigators engaged" [title/abstract:~2] OR "investigators engagement" [title/abstract:~2] OR "investigators engaging" [title/abstract:~2] OR "investigators experiences" [title/abstract:~2] OR "investigators involved" [title/abstract:~2] OR "investigators involvement" [title/abstract:~2] OR "investigators participated" [title/abstract:~2] OR "investigators participating" [title/abstract:~2] OR "investigators participation" [title/abstract:~2] OR "investigators perceptions" [title/abstract:~2] OR "investigators responded" [title/abstract:~2] OR "investigators responding" [title/abstract:~2] OR "investigators responses" [title/abstract: ~2] OR "physician-scientist engaged" [title/abstract:~2] OR "physician-scientist engagement" [title/abstract:~2] OR "physician-scientist engaging" [title/abstract:~2] OR "physician-scientist experiences" [title/abstract:~2] OR "physician-scientist involved" [title/abstract:~2] OR "physician-scientist involvement" [title/abstract:~2] OR "physician-scientist participated" [title/abstract:~2] OR "physician-scientist participating" [title/abstract:~2] OR "physician-scientist participation" [title/abstract:~2] OR "physician-scientist perceptions" [title/abstract:~2] OR "physician-scientist responded" [title/abstract:~2] OR "physician-scientist responding" [title/abstract:~2] OR "physician-scientist responses" [title/abstract: ~2] OR "physician-scientist's experiences" [title/abstract:~2] OR "physician-scientist's perceptions" [title/abstract:~2] OR "physician-scientist's responses" [title/abstract: ~2] OR "physician-scientists engaged" [title/abstract:~2] OR "physician-scientists engagement" [title/abstract:~2] OR "physician-scientists engaging" [title/abstract:~2] OR "physician-scientists experiences" [title/abstract:~2] OR "physician-scientists involved" [title/abstract:~2] OR "physician-scientists involvement" [title/abstract:~2] OR "physician-scientists participated" [title/abstract:~2] OR "physician-scientists participating" [title/abstract:~2] OR "physician-scientists participation" [title/abstract:~2] OR "physician-scientists perceptions" [title/abstract:~2] OR "physician-scientists responded" [title/abstract:~2] OR "physician-scientists responding" [title/abstract:~2] OR "physician-scientists responses" [title/abstract: ~2] OR "professor engaged" [title/abstract:~2] OR "professor engagement" [title/abstract:~2] OR "professor engaging" [title/abstract:~2] OR "professor experiences" [title/abstract:~2] OR "professor experiences" [title/abstract:~2] OR "professor involved" [title/abstract:~2] OR "professor involvement" [title/abstract:~2] OR "professor participated" [title/abstract:~2] OR "professor participating" [title/abstract:~2] OR "professor participation" [title/abstract:~2] OR "professor perceptions" [title/abstract:~2] OR "professor perceptions" [title/abstract:~2] OR "professor responded" [title/abstract:~2] OR "professor responding" [title/abstract:~2] OR "professor responses" [title/abstract: ~2] OR "professor responses" [title/abstract: ~2] OR "professor's experiences" [title/abstract:~2] OR "professor's perceptions" [title/abstract:~2] OR "professor's responses" [title/abstract: ~2] OR "professors engaged" [title/abstract:~2] OR "professors engagement" [title/abstract:~2] OR "professors engaging" [title/abstract:~2] OR "professors experiences" [title/abstract:~2] OR "professors experiences" [title/abstract:~2] OR "professors involved" [title/abstract:~2] OR "professors involvement" [title/abstract:~2] OR "professors participated" [title/abstract:~2] OR "professors participating" [title/abstract:~2] OR "professors participation" [title/abstract:~2] OR "professors perceptions" [title/abstract:~2] OR "professors perceptions" [title/abstract:~2] OR "professors responded" [title/abstract:~2] OR "professors responding" [title/abstract:~2] OR "professors responses" [title/abstract: ~2] OR "professors responses" [title/abstract:~2] OR "project team engaged" [title/abstract:~2] OR "project team engagement" [title/abstract:~2] OR "project team engaging" [title/abstract:~2] OR "project team experiences" [title/abstract:~2] OR "project team involved" [title/abstract:~2] OR "project team involvement" [title/abstract:~2] OR "project team participated" [title/abstract:~2] OR "project team participating" [title/abstract:~2] OR "project team participation" [title/abstract:~2] OR "project team perceptions" [title/abstract:~2] OR "project team responded" [title/abstract:~2] OR "project team responding" [title/abstract:~2] OR "project team responses" [title/abstract: ~2] OR "project team's experiences" [title/abstract:~2] OR "project team's perceptions" [title/abstract:~2] OR "project team's responses" [title/abstract: ~2] OR "project teams engaged" [title/abstract:~2] OR "project teams engagement" [title/abstract:~2] OR "project teams engaging" [title/abstract:~2] OR "project teams experiences" [title/abstract:~2] OR "project teams involved" [title/abstract:~2] OR "project teams involvement" [title/abstract:~2] OR "project teams participated" [title/abstract:~2] OR "project teams participating" [title/abstract:~2] OR "project teams participation" [title/abstract:~2] OR "project teams perceptions" [title/abstract:~2] OR "project teams responded" [title/abstract:~2] OR "project teams responding" [title/abstract:~2] OR "project teams responses" [title/abstract: ~2] OR "research assistant engaged" [title/abstract:~2] OR "research assistant engagement" [title/abstract:~2] OR "research assistant engaging" [title/abstract:~2] OR "research assistant experiences" [title/abstract:~2] OR "research assistant involved" [title/abstract:~2] OR "research assistant involvement" [title/abstract:~2] OR "research assistant participated" [title/abstract:~2] OR "research assistant participating" [title/abstract:~2] OR "research assistant participation" [title/abstract:~2] OR "research assistant perceptions" [title/abstract:~2] OR "research assistant responded" [title/abstract:~2] OR "research assistant responding" [title/abstract:~2] OR "research assistant responses" [title/abstract: ~2] OR "research assistant's experiences" [title/abstract:~2] OR "research assistant's perceptions" [title/abstract:~2] OR "research assistant's responses" [title/abstract: ~2] OR "research assistants engaged" [title/abstract:~2] OR "research assistants engagement" [title/abstract:~2] OR "research assistants engaging" [title/abstract:~2] OR "research assistants experiences" [title/abstract:~2] OR "research assistants involved" [title/abstract:~2] OR "research assistants involvement" [title/abstract:~2] OR "research assistants participated" [title/abstract:~2] OR "research assistants participating" [title/abstract:~2] OR "research assistants participation" [title/abstract:~2] OR "research assistants perceptions" [title/abstract:~2] OR "research assistants responded" [title/abstract:~2] OR "research assistants responding" [title/abstract:~2] OR "research assistants responses" [title/abstract: ~2] OR "research faculty engaged" [title/abstract:~2] OR "research faculty engagement" [title/abstract:~2] OR "research faculty engaging" [title/abstract:~2] OR "research faculty experiences" [title/abstract:~2] OR "research faculty involved" [title/abstract:~2] OR "research faculty involvement" [title/abstract:~2] OR "research faculty participated" [title/abstract:~2] OR "research faculty participating" [title/abstract:~2] OR "research faculty participation" [title/abstract:~2] OR "research faculty perceptions" [title/abstract:~2] OR "research faculty responded" [title/abstract:~2] OR "research faculty responding" [title/abstract:~2] OR "research faculty responses" [title/abstract: ~2] OR "research faculty's engaged" [title/abstract:~2] OR "research faculty's engagement" [title/abstract:~2] OR "research faculty's engaging" [title/abstract:~2] OR "research faculty's experiences" [title/abstract:~2] OR "research faculty's involved" [title/abstract:~2] OR "research faculty's involvement" [title/abstract:~2] OR "research faculty's participated" [title/abstract:~2] OR "research faculty's participating" [title/abstract:~2] OR "research faculty's participation" [title/abstract:~2] OR "research faculty's perceptions" [title/abstract:~2] OR "research faculty's responded" [title/abstract:~2] OR "research faculty's responding" [title/abstract:~2] OR "research faculty's responses" [title/abstract: ~2] OR "research personnel engaged" [title/abstract:~2] OR "research personnel engagement" [title/abstract:~2] OR "research personnel engaging" [title/abstract:~2] OR "research personnel experiences" [title/abstract:~2] OR "research personnel involved" [title/abstract:~2] OR "research personnel involvement" [title/abstract:~2] OR "research personnel participated" [title/abstract:~2] OR "research personnel participating" [title/abstract:~2] OR "research personnel participation" [title/abstract:~2] OR "research personnel perceptions" [title/abstract:~2] OR "research personnel responded" [title/abstract:~2] OR "research personnel responding" [title/abstract:~2] OR "research personnel responses" [title/abstract: ~2] OR "research personnel's experiences" [title/abstract:~2] OR "research personnel's perceptions" [title/abstract:~2] OR "research personnel's responses" [title/abstract: ~2] OR "research staff engaged" [title/abstract:~2] OR "research staff engagement" [title/abstract:~2] OR "research staff engaging" [title/abstract:~2] OR "research staff experiences" [title/abstract:~2] OR "research staff involved" [title/abstract:~2] OR "research staff involvement" [title/abstract:~2] OR "research staff participated" [title/abstract:~2] OR "research staff participating" [title/abstract:~2] OR "research staff participation" [title/abstract:~2] OR "research staff perceptions" [title/abstract:~2] OR "research staff responded" [title/abstract:~2] OR "research staff responding" [title/abstract:~2] OR "research staff responses" [title/abstract: ~2] OR "research staff's experiences" [title/abstract:~2] OR "research staff's perceptions" [title/abstract:~2] OR "research staff's responses" [title/abstract: ~2] OR "research staffs engaged" [title/abstract:~2] OR "research staffs engagement" [title/abstract:~2] OR "research staffs engaging" [title/abstract:~2] OR "research staffs experiences" [title/abstract:~2] OR "research staffs involved" [title/abstract:~2] OR "research staffs involvement" [title/abstract:~2] OR "research staffs participated" [title/abstract:~2] OR "research staffs participating" [title/abstract:~2] OR "research staffs participation" [title/abstract:~2] OR "research staffs perceptions" [title/abstract:~2] OR "research staffs responded" [title/abstract:~2] OR "research staffs responding" [title/abstract:~2] OR "research staffs responses" [title/abstract: ~2] OR "research team engaged" [title/abstract:~2] OR "research team engagement" [title/abstract:~2] OR "research team engaging" [title/abstract:~2] OR "research team experiences" [title/abstract:~2] OR "research team involved" [title/abstract:~2] OR "research team involvement" [title/abstract:~2] OR "research team participated" [title/abstract:~2] OR "research team participating" [title/abstract:~2] OR "research team participation" [title/abstract:~2] OR "research team perceptions" [title/abstract:~2] OR "research team responded" [title/abstract:~2] OR "research team responding" [title/abstract:~2] OR "research team responses" [title/abstract: ~2] OR "research team's experiences" [title/abstract:~2] OR "research team's perceptions" [title/abstract:~2] OR "research team's responses" [title/abstract: ~2] OR "research teams engaged" [title/abstract:~2] OR "research teams engagement" [title/abstract:~2] OR "research teams engaging" [title/abstract:~2] OR "research teams experiences" [title/abstract:~2] OR "research teams involved" [title/abstract:~2] OR "research teams involvement" [title/abstract:~2] OR "research teams participated" [title/abstract:~2] OR "research teams participating" [title/abstract:~2] OR "research teams participation" [title/abstract:~2] OR "research teams perceptions" [title/abstract:~2] OR "research teams responded" [title/abstract:~2] OR "research teams responding" [title/abstract:~2] OR "research teams responses" [title/abstract: ~2] OR "research worker engaged" [title/abstract:~2] OR "research worker engagement" [title/abstract:~2] OR "research worker engaging" [title/abstract:~2] OR "research worker experiences" [title/abstract:~2] OR "research worker involved" [title/abstract:~2] OR "research worker involvement" [title/abstract:~2] OR "research worker participated" [title/abstract:~2] OR "research worker participating" [title/abstract:~2] OR "research worker participation" [title/abstract:~2] OR "research worker perceptions" [title/abstract:~2] OR "research worker responded" [title/abstract:~2] OR "research worker responding" [title/abstract:~2] OR "research worker responses" [title/abstract: ~2] OR "research workers engaged" [title/abstract:~2] OR "research workers engagement" [title/abstract:~2] OR "research workers engaging" [title/abstract:~2] OR "research workers experiences" [title/abstract:~2] OR "research workers involved" [title/abstract:~2] OR "research workers involvement" [title/abstract:~2] OR "research workers participated" [title/abstract:~2] OR "research workers participating" [title/abstract:~2] OR "research workers participation" [title/abstract:~2] OR "research workers perceptions" [title/abstract:~2] OR "research workers responded" [title/abstract:~2] OR "research workers responding" [title/abstract:~2] OR "research workers responses" [title/abstract: ~2] OR "research workforce engaged" [title/abstract:~2] OR "research workforce engagement" [title/abstract:~2] OR "research workforce engaging" [title/abstract:~2] OR "research workforce experiences" [title/abstract:~2] OR "research workforce involved" [title/abstract:~2] OR "research workforce involvement" [title/abstract:~2] OR "research workforce participated" [title/abstract:~2] OR "research workforce participating" [title/abstract:~2] OR "research workforce participation" [title/abstract:~2] OR "research workforce perceptions" [title/abstract:~2] OR "research workforce responded" [title/abstract:~2] OR "research workforce responding" [title/abstract:~2] OR "research workforce responses" [title/abstract: ~2] OR "research workforce's experiences" [title/abstract:~2] OR "research workforce's perceptions" [title/abstract:~2] OR "research workforce's responses" [title/abstract: ~2] OR "researcher engaged" [title/abstract:~2] OR "researcher engagement" [title/abstract:~2] OR "researcher engaging" [title/abstract:~2] OR "researcher experiences" [title/abstract:~2] OR "researcher involved" [title/abstract:~2] OR "researcher involvement" [title/abstract:~2] OR "researcher participated" [title/abstract:~2] OR "researcher participating" [title/abstract:~2] OR "researcher participation" [title/abstract:~2] OR "researcher perceptions" [title/abstract:~2] OR "researcher responded" [title/abstract:~2] OR "researcher responding" [title/abstract:~2] OR "researcher responses" [title/abstract: ~2] OR "researcher's experiences" [title/abstract:~2] OR "researcher's perceptions" [title/abstract:~2] OR "researcher's responses" [title/abstract: ~2] OR "researchers engaged" [title/abstract:~2] OR "researchers engagement" [title/abstract:~2] OR "researchers engaging" [title/abstract:~2] OR "researchers experiences" [title/abstract:~2] OR "researchers involved" [title/abstract:~2] OR "researchers involvement" [title/abstract:~2] OR "researchers participated" [title/abstract:~2] OR "researchers participating" [title/abstract:~2] OR "researchers participation" [title/abstract:~2] OR "researchers perceptions" [title/abstract:~2] OR "researchers responded" [title/abstract:~2] OR "researchers responding" [title/abstract:~2] OR "researchers responses" [title/abstract:~2] OR "scientist engaged" [title/abstract:~2] OR "scientist engagement" [title/abstract:~2] OR "scientist engaging" [title/abstract:~2] OR "scientist experiences" [title/abstract:~2] OR "scientist involved" [title/abstract:~2] OR "scientist involvement" [title/abstract:~2] OR "scientist participated" [title/abstract:~2] OR "scientist participating" [title/abstract:~2] OR "scientist participation" [title/abstract:~2] OR "scientist perceptions" [title/abstract:~2] OR "scientist responded" [title/abstract:~2] OR "scientist responding" [title/abstract:~2] OR "scientist responses" [title/abstract: ~2] OR "scientist's experiences" [title/abstract:~2] OR "scientist's perceptions" [title/abstract:~2] OR "scientist's responses" [title/abstract: ~2] OR "scientists engaged" [title/abstract:~2] OR "scientists engagement" [title/abstract:~2] OR "scientists engaging" [title/abstract:~2] OR "scientists experiences" [title/abstract:~2] OR "scientists involved" [title/abstract:~2] OR "scientists involvement" [title/abstract:~2] OR "scientists participated" [title/abstract:~2] OR "scientists participating" [title/abstract:~2] OR "scientists participation" [title/abstract:~2] OR "scientists perceptions" [title/abstract:~2] OR "scientists responded" [title/abstract:~2] OR "scientists responding" [title/abstract:~2] OR "scientists responses" [title/abstract: ~2] OR "study personnel engaged" [title/abstract:~2] OR "study personnel engagement" [title/abstract:~2] OR "study personnel engaging" [title/abstract:~2] OR "study personnel experiences" [title/abstract:~2] OR "study personnel involved" [title/abstract:~2] OR "study personnel involvement" [title/abstract:~2] OR "study personnel participated" [title/abstract:~2] OR "study personnel participating" [title/abstract:~2] OR "study personnel participation" [title/abstract:~2] OR "study personnel perceptions" [title/abstract:~2] OR "study personnel responded" [title/abstract:~2] OR "study personnel responding" [title/abstract:~2] OR "study personnel responses" [title/abstract: ~2] OR "study staff engaged" [title/abstract:~2] OR "study staff engagement" [title/abstract:~2] OR "study staff engaging" [title/abstract:~2] OR "study staff experiences" [title/abstract:~2] OR "study staff involved" [title/abstract:~2] OR "study staff involvement" [title/abstract:~2] OR "study staff participated" [title/abstract:~2] OR "study staff participating" [title/abstract:~2] OR "study staff participation" [title/abstract:~2] OR "study staff perceptions" [title/abstract:~2] OR "study staff responded" [title/abstract:~2] OR "study staff responding" [title/abstract:~2] OR "study staff responses" [title/abstract: ~2] OR "study staffs engaged" [title/abstract:~2] OR "study staffs engagement" [title/abstract:~2] OR "study staffs engaging" [title/abstract:~2] OR "study staffs involved" [title/abstract:~2] OR "study staffs involvement" [title/abstract:~2] OR "study staffs participated" [title/abstract:~2] OR "study staffs participating" [title/abstract:~2] OR "study staffs participation" [title/abstract:~2] OR "study staffs responded" [title/abstract:~2] OR "study staffs responding" [title/abstract:~2] OR "study team engaged" [title/abstract:~2] OR "study team engagement" [title/abstract:~2] OR "study team engaging" [title/abstract:~2] OR "study team experiences" [title/abstract:~2] OR "study team involved" [title/abstract:~2] OR "study team involvement" [title/abstract:~2] OR "study team participated" [title/abstract:~2] OR "study team participating" [title/abstract:~2] OR "study team participation" [title/abstract:~2] OR "study team perceptions" [title/abstract:~2] OR "study team responded" [title/abstract:~2] OR "study team responding" [title/abstract:~2] OR "study team responses" [title/abstract: ~2] OR "study teams engaged" [title/abstract:~2] OR "study teams engagement" [title/abstract:~2] OR "study teams engaging" [title/abstract:~2] OR "study teams experiences" [title/abstract:~2] OR "study teams involved" [title/abstract:~2] OR "study teams involvement" [title/abstract:~2] OR "study teams participated" [title/abstract:~2] OR "study teams participating" [title/abstract:~2] OR "study teams participation" [title/abstract:~2] OR "study teams perceptions" [title/abstract:~2] OR "study teams responded" [title/abstract:~2] OR "study teams responding" [title/abstract:~2] OR "study teams responses" [title/abstract: ~2] OR "trialist engaged" [title/abstract:~2] OR "trialist engagement" [title/abstract:~2] OR "trialist engaging" [title/abstract:~2] OR "trialist experiences" [title/abstract:~2] OR "trialist involved" [title/abstract:~2] OR "trialist involvement" [title/abstract:~2] OR "trialist participated" [title/abstract:~2] OR "trialist participating" [title/abstract:~2] OR "trialist participation" [title/abstract:~2] OR "trialist perceptions" [title/abstract:~2] OR "trialist responded" [title/abstract:~2] OR "trialist responding" [title/abstract:~2] OR "trialist responses" [title/abstract: ~2] OR "trialists engaged" [title/abstract:~2] OR "trialists engagement" [title/abstract:~2] OR "trialists engaging" [title/abstract:~2] OR "trialists experiences" [title/abstract:~2] OR "trialists involved" [title/abstract:~2] OR "trialists involvement" [title/abstract:~2] OR "trialists participated" [title/abstract:~2] OR "trialists participating" [title/abstract:~2] OR "trialists participation" [title/abstract:~2] OR "trialists perceptions" [title/abstract:~2] OR "trialists responded" [title/abstract:~2] OR "trialists responding" [title/abstract:~2] OR "trialists responses" [title/abstract: ~2]) OR (("clinician-scientist*" [title/abstract] OR "investigator*" [title/abstract] OR "physician-scientist*" [title/abstract] OR "professor*" [title/abstract] OR "project team*" [title/abstract] OR "research assistant*" [title/abstract] OR "research facult*" [title/abstract] OR "research personnel" [mesh] OR "research personnel*" [title/abstract] OR "research staff*" [title/abstract] OR "research work*" [title/abstract] OR "researcher*" [title/abstract] OR "scientist*" [title/abstract] OR "study personnel" [title/abstract] OR "study staff*" [title/abstract] OR "study team*" [title/abstract] OR "trialist*" [title/abstract]) AND ("meta-research" [title/abstract] OR "metaresearch" [title/abstract] OR "meta-science" [title/abstract] OR "metascience" [title/abstract] OR "research on research*" [title/abstract] OR "research personnel/psychology" [mesh] OR "research personnel/statistics and numerical data" [mesh] OR "research personnel/trends" [mesh] OR "research-on-research*" [title/abstract]))) AND ("english" [language])
