# Supplementary material for: Identifying meta-research with researchers as study subjects: Protocol for a scoping review
Source: PLoS One. 2024 May 20;19(5):e0303905. doi: 10.1371/journal.pone.0303905 (PMC11104640; doi:10.1371/journal.pone.0303905)
Supplement: Attachment — Submitted filename: Response to reviewers.docx [file pone.0303905.s004.docx]

**Response to reviewers**

**Editor**

1. **Please ensure that your manuscript meets PLOS ONE's style requirements, including those for file naming.**

Complete

2. **In your cover letter, please confirm that the** **research you have described in your manuscript, including participant recruitment, data collection, modification, or processing, has not started and will not start until after your paper has been accepted to the journal (assuming data need to be collected or participants recruited specifically for your study). In order to proceed with your submission, you must provide confirmation.**

Complete

3. **Please expand the acronym “NIA” (as indicated in your financial disclosure) so that it states the name of your funders in full. This information should be included in your cover letter; we will change the online submission form on your behalf.**

Complete

4. **Please remove any funding-related text from the manuscript and let us know how you would like to update your Funding Statement. Currently, your Funding Statement reads as follows: "ES received funding from the NIA (https://www.nia.nih.gov/) (Grant--1K01AG075169-01A1). The funders did not and will not have a role in study design, data collection and analysis, decision to publish, or preparation of the manuscript." Please include your amended statements within your cover letter; we will change the online submission form on your behalf.**

Complete—no changes

5. **Please include captions for your Supporting Information files at the end of your manuscript, and update any in-text citations to match accordingly.**

Complete

6. **Please upload copies of the completed PRISMA checklist as Supporting Information with a file name “****PRISMA checklist”.**

Complete

7. **The second reviewer has not seen that you submitted a Registered Report Protocol. Thus, please ignore his/her corresponding comments.**

Those specific comments have been disregarded.

**Reviewer #1**

1. **p. 12 l. 280 a word seems to be missing in this phrase: "studies that would eligibility criteria for inclusion in the scoping review."**

Thank you for identifying this error. We have added to the sentence so it now reads: “…studies that would fit eligibility criteria for inclusion in the scoping review.”

**Reviewer #2**

We thank the reviewer for their close read of the manuscript and interest in the topic presented. As per the editor comments regarding the Registered Report Protocol, we are not addressing the comments specific to publication criteria.

1. **Literature: The manuscript does not reflect literature from “Quantitative Science Studies”, “Science of Science”, or “Research on Research”, which are mainly concerned with this topic, also in meta-perspective. The revised manuscript should demonstrate the need for meta-research from the existing literature.**

The first paragraph of the introduction seeks to discuss the scoping reviews that have been performed on meta-research topics (ln 60-64). We have attempted to clarify that our term of “meta-research” is research on research and have now placed clarification at the first instance of “meta-research” use. Now stating, “Specifically in the field of meta-research (research on research)…”

2. **Interdisciplinarity: Researchers as objects of study can be examined from very different perspectives, such as psychological (e.g. motivation), medical (e.g. influence of testosterone), statistical and sociological. I wonder whether scoping reviews of this kind do not also require special expertise in a field and special questions in order to be able to draw conclusions from them. However, this is not discussed in the draft.**

We agree that the study of researchers has the potential to be a very interdisciplinary body of literature. As a scoping review, the aim of this protocol is to create an overview of the literature available in this topic (research on researchers) including a description of the types of research—e.g. psychological, epidemiological, etc.—being performed in this population. In the protocol section “Stage 4: Charting the data” (ln 247-256) we specify the types of data to be extracted in the review and include “data collection methods and types of data used” to capture the type of research being performed. We believe that this scoping review does not require an expansively interdisciplinary to perform this summarization function. However, if future reviews are performed that include the interpretation of the results, a more interdisciplinary team will be considered.

3. **Lack of differentiation to meta-analysis: The proposed steps are more or less identical to those of a meta-analysis. I wonder if there is a big difference between scoping reviews and meta-analysis or ordinary reviews.**

The search methods used in a scoping review are very similar to a meta-analysis as both seek to use rigorous and replicable search strategies, however, the type of research question being answered differs between these types of reviews. A scoping review seeks to present an overview of a body of literature pertaining to a broad topic. A systematic review (including meta-analyses) attempts to collate empirical evidence pertaining to a focused research question. We have specified in our Methods section the scoping review methods guidelines that were followed, stating: (ln 117-119) “The scoping review protocol is guided by the methodological frameworks established by the Joanna Briggs Institute (JBI) scoping review methodology and by Arksey and O' Malley (2005).”

Arksey H, O'Malley L. Scoping studies: towards a methodological framework. International Journal of Social Research Methodology. 2005;8(1):19-32.

Peters MDJ, Godfrey C, McInerney P, Khalil H, Larsen P, Marnie C, et al. Best practice guidance and reporting items for the development of scoping review protocols. JBI Evid Synth. 2022;20(4):953-68.

4. **Reference style: According to the “Vancouver” style, PLOS One requires the following: “In the text, cite the reference number in square brackets (e.g., “We used the techniques developed by our colleagues [19] to analyze the data”). PLOS uses the numbered citation (citation-sequence) method and first six authors, et al.” (**[**Link**](https://journals.plos.org/plosone/s/submission-guidelines#loc-references)**).**

The formatting of the paper has been updated to reflect the style requirements of PlosOne.
