# Supplementary material for: Identifying meta-research with researchers as study subjects: Protocol for a scoping review
Source: PLoS One. 2024 May 20;19(5):e0303905. doi: 10.1371/journal.pone.0303905 (PMC11104640; doi:10.1371/journal.pone.0303905)
Supplement: Attachment — Submitted filename: Response to PLOS Reviewers 3.29.24.docx [file pone.0303905.s005.docx]

**Response to Reviewers**

1. **Have the authors described where all data underlying the findings will be made available when the study is complete?
   Reviewer #2: No**

We have updated the Data Availability Statement for the study to note that we intend to make publicly available all relevant data from all stages of scoping review, including the development of the scoping review protocol and the conduct of the scoping review. Data on the testing of the preliminary search strategy will be made available as a Supporting Information file included with the manuscript. Once the scoping review is conducted, data on included and excluded citations will be included with as a Supporting Information file

2. **Meta-research: The introduction is essentially based on a presentation of a new method, meta-research, but the real issue is the question of researchers as subjects of scientific studies and how research in this area can be synthesised. Meta-research is one of several possible methods, along with meta-analysis and systematic review, to address this question. I recommend starting with the research gap and then presenting meta-research as an approach to answering the question.**

Within this protocol we are using the definition of meta-research as a research discipline rather than a specific research method (i.e. the research method meta-analysis). Meta-research is a discipline designed to study research itself and its practices and the objective is to understand and improve how we perform, communicate, verify, evaluate, incentivize, and support research. The purpose of this study is to identify how researchers are studied as research subjects. We are thus using the method of scoping review to conduct this meta-research study.

3. **Motivating the research question: In my view there is no sufficient derivation of the research question from the research literature (e.g., quantitative scientific studies) on questions where the researchers themselves were made the subject of the research. Reference is made to a "body of literature", but no literature is cited, although a body of empirical literature must be assumed. The question remains as to the scientific background against which the results in the registered report can be interpreted and used.**

We appreciate the importance of presenting the body of literature to the reader, however there are no readily citable references that encapsulate, summarize, or discuss as a whole (or in part) the body of literature where researchers themselves are the study subjects. A citation to this literature would simply be a collection of example studies. This gap in the literature is specifically being addressed by the scoping review proposed in this protocol.

Understanding this limitation, to make the statements alluding to the “body of literature” better grounded, we have revised the text to discuss the utility of meta-research more generally, citing the literature in that area and calling out research with researchers themselves as research subjects as an example. Specifically, we now state:

(p.4, ln 88-93) Just as the examination of research practices in other areas of meta-research can help disseminate efficient and effective research policies and to identify and abandon wasteful ones, insights from the body of literature with researchers as the study subjects can illuminate how to better support researchers, enhance the development of research capacity, and advance the methods used to engage and study this population.

4. **Registered report protocol: I wonder why this study was submitted as a registered report protocol. Registered report protocols are useful if you have formulated a clear research question, hypotheses, and an experimental design to test the hypotheses. This makes it possible to publish results in the subsequent study that contradict the original hypotheses. In the study plan presented no hypotheses are formulated; it is an exploratory study. A potential risk of bias is not recognisable. The plan for the data analysis and reporting is vague (e.g., tabular reports, thematic analyses, narrative summaries). Results of a preliminary study with a bibliographic database are not reported (e.g., potential number of studies, text-mining for key words). A sufficient justification for the type of article is missing.**

We have submitted this scoping review protocol as a Registered Report Protocol because it is a protocol for a proposed study for which data has not yet been collected (except for testing of the proposed search strategy). As described in the PLOS One submission guidelines, a Registered Report Protocol is: “an article describing the study design, rationale, timeline, proposed methodology for data collection and analysis, and where applicable ethical approval for the work. Registered Report Protocols report the study proposal prior to conducting experiments, data collection patient recruitment, and they undergo peer review to ensure that the planned research will meet *PLOS ONE*’s publication criteria.” This scoping review protocol falls within the purview of the Registered Report Protocol. However, because PLOS One has published scoping review protocols both as Registered Report Protocols and as Study Protocols, we can submit the protocol as a Study Protocol instead if the reviewer thinks that is more appropriate than Registered Report Protocol.

5. **Component B: It was stated (p. 9) that “These names are then combined with a series of keywords identifying actions performed by researchers as study subjects”. I wonder where these keywords (e.g., engaged, …) come from. Important verbs as for example “investigate” or “conduct” are missing. The revision should give some reasons for the selection of keywords for actions.**

The intention of these key terms is to identify ways in which researchers tend to be described in the context of being themselves the subjects of research. The keywords were identified through listing terms commonly found in the titles and abstracts of known studies that met eligibility criteria for the scoping review. “Investigate” and “conduct” were not selected to be used as they do not specifically identify ways in which researchers tend to be described in the context of being themselves the subjects of research.

6. **Funding: The financial disclosure stated that "funding was received from the National Institute of Aging (NIA)". This raises the question of who will fund the subsequent study.**

The conduct of the scoping review will be funded with the same funding.
